# Maternal Health of Female Sex Workers in Six Low- and Middle-Income Countries

**DOI:** 10.2147/IJWH.S550242

**Published:** 2026-03-31

**Authors:** Heather Thompson, Brian Willis, Swarna D S Weerasinghe, Emily K Perttu, Esther Anne Chin, Wendy L Macias-Konstantopoulos

**Affiliations:** 1 Department of OBGYN, McMaster University, Hamilton, Canada; 2 Department of OBGYN, Brightshores Health System, Owen Sound, Canada; 3 Global Health Promise, Portland, OR, USA; 4 Department of Community Health and Epidemiology, Dalhousie University, Halifax, Canada; 5 Center for Social Justice and Health Equity, Department of Emergency Medicine, Massachusetts General Hospital; Harvard Medical School, Boston, MA, USA

**Keywords:** female sex workers, maternal health, sex work, public health, global perspectives, service needs, antenatal care access

## Abstract

**Purpose:**

The purpose of this study was to examine factors that impact maternal health, including antenatal care and skilled birth attendance, work behavior, and substance use during pregnancy among female sex workers (FSW) across six low- and middle-income countries (LMIC). The added knowledge may inform programs, policies, and funding on a level equivalent to those for HIV prevention to reduce the high numbers of maternal deaths among this population.

**Methods:**

This was an exploratory mixed methods study. Quantitative data were gathered from cross-sectional surveys completed by 1352 FSW. The proxy respondent methodology was used to identify behaviors and characteristics related to maternal health in pregnancy among FSW. Descriptive statistics were used to summarize quantitative survey responses, and text analytics to summarize answers to open-ended questions.

**Results:**

An estimated 86.1% of FSW were reported to be mothers, but access to antenatal care (ANC) was reported to be only 28.7%. Barriers to ANC included cost, stigma, and accessibility. In some countries the majority of FSW were reported to give birth in brothels. Nearly a quarter (24.4%) of FSW were reported to work until the onset of labor, with most (75.9%) returning to work less than a month postpartum. Consumption of alcohol and other substances during pregnancy was reportedly very high (79.0% and 65.1% respectively), and 70.6% of FSW were reported to experience depression during the postpartum period. Participants expressed the need for respectful FSW-sensitized prenatal, intrapartum, and postpartum care at a location where they felt comfortable.

**Conclusion:**

This exploratory study revealed multiple risk factors experienced by pregnant FSW that may contribute to high numbers of maternal deaths reported among FSW, including poor access to affordable, quality maternal healthcare, and lack of service programs to address social determinants of health.

## Plain Language Summary

This study looked at pregnancy-related healthcare among female sex workers (FSW) in six low- and middle-income countries (LMIC). It explored factors that could affect the risk of complications during pregnancy and delivery, such as prenatal appointment attendance, delivering with a skilled provider, substance use in pregnancy, maternal depression, and physically demanding work through late gestation. Using a structured questionnaire, we asked FSW participants about these practices among their peers. There was some variation between countries, but overall we found that FSW tended to have low attendance at prenatal appointments, often had babies in places without skilled medical care, reported high rates of drug and alcohol use during pregnancy, and often worked until the late in pregnancy and returned to work shortly after the baby was born. The FSW expressed a need for quality and affordable pregnancy-related services where the care is respectful and sensitized to the unique needs and pressures of FSW mothers.

## Introduction

Female sex workers (FSW) are among the most marginalized and understudied populations in public health and face multiple structural vulnerabilities.^1^ Sex work is stigmatized and illegal in many countries, thereby limiting FSWs’ access to healthcare and rendering them vulnerable to a variety of adverse health outcomes.^2^ Research-based evidence, as well as preventive health programming, almost exclusively focuses on FSWs’ heightened risk of contracting and transmitting HIV and other sexually transmitted infections, and rarely addresses the full spectrum of maternal health, including healthcare related needs, health behaviours, and barriers to accessibility.

There is a scarcity of research on FSWs’ reproductive and maternal health outcomes^3^,^4^ even as the vast majority of FSWs become pregnant and have children.^5–7^ Up to 90% of FSWs in developing countries are mothers,^5–7^ but they generally receive limited or no sexual and reproductive health services.^8–10^

Maternal deaths account for a third of the world’s premature mortality, and 90% of these deaths are preventable.^11^ Much global maternal mortality is due to lack of appropriate prenatal and intrapartum healthcare.^12^ Multi-country FSW mortality data suggest that the majority of deaths among FSW are from maternal causes (62.5%), with 35.5% of these deaths are related to complications from unsafe abortion and an additional 16.6% deaths from other maternal causes, such as hemorrhage.^13^ The WHO has identified that appropriate antenatal care, intrapartum care, and postpartum care are key to preventing maternal mortality while improving maternal and newborn health.^11^

Quality antenatal care (ANC) is a vital component of maternal healthcare and critical for health promotion and prevention, screening, and diagnosis of disease, all of which improve maternal and fetal health, and decrease mother-to-child transmission of HIV.^14^ ANC reduces maternal and perinatal mortality by identifying women at risk of pregnancy-related complications so they can be managed appropriately, while also allowing for prevention and management of concurrent illnesses that account for indirect maternal deaths, such as HIV.^11^

WHO recommends a minimum of eight ANC visits in pregnancy to optimize fetal and maternal health and to facilitate delivery of complementary health information, screening, or treatment for tuberculosis, malaria, and HIV.^11^ The antenatal period is a critical time for educating mothers-to-be about nutrition, prevention of mother-to-child HIV transmission, breastfeeding, reasons to seek care, and the importance of intrapartum care with a skilled birth attendant.^15^ Additionally, ANC can be a gateway to other healthcare services such as mental health counseling and postpartum family planning.^7^ Receiving ANC has also been shown to increase the likelihood of having a skilled birthing attendant, which reduces maternal and neonatal mortality.^16^,^17^ Notably, the WHO increased its recommendation from four ANC visits to eight, as research found that additional appointments decreased perinatal mortality, demonstrating the vital importance of ANC in achieving a safe pregnancy and delivery.^11^

Antenatal care is important for all pregnant women, but especially for FSW due to their increased risk of adverse pregnancy outcomes resulting from a high burden of HIV, poor nutritional status, and exposure to violence.^5^,^18^ Up to two-thirds of FSWs live with HIV, and most are mothers.^5^,^6^ Diagnosing HIV antenatally is critical as the risk of vertical HIV transmission can be minimized if women are started on antiretrovirals before 14 weeks gestational age.^19^

There is limited knowledge about ANC-seeking behavior among pregnant FSWs. Many FSW do not realize that they are pregnant until the second or third trimester, narrowing the time frame in which they can seek antenatal care or safe abortion.^6^ One study reported the average gestational age at first antenatal visit for FSW was about 20 weeks, well past the 12-week initial visit recommended by the WHO,^5^,^15^ and the 14-week mark to start antiretrovirals to reduce mother-to-child transmission.

Skilled birth attendance (SBA) refers to the presence of a trained health professional during labour, delivery, and early postpartum.^20^ It also includes an “enabling environment” where there are appropriate resources to deliver a baby safely, and transport the mother to higher level of care if needed.^20^ With skilled attendants, complications can be identified early and managed; this is critical as two-thirds of maternal deaths occur during the birthing process, and it can be difficult to predict who will encounter such difficulties.^20^ Data on FSW delivery practices is very limited.^21^

Mental health problems, such as anxiety and depression, are an important global cause of disability, and mortality from suicide is strongly associated with mental health conditions.^22^ Postpartum depression is common, especially among women with previous depression.^23^ Due to multiple overlapping factors including stigma, poverty, and violence, FSW are especially vulnerable to mental health issues, and have higher rates of mental health issues than the general population.^22^ Despite the high rates of depression among FSW, and the known increased vulnerability of postpartum women to depression, there have been almost no studies to our knowledge on the prevalence of depression or suicide among pregnant or postpartum FSW. A recent multi-LMIC study found that 62% of all FSW suicides were maternal suicides, occurring during pregnancy or the first-year postpartum.^13^ Additionally, a subsequent analysis found that the majority (58%) of these FSW suicides occurred in the antepartum period during pregnancy, while 20% were in women within 2 months of delivery, highlighting the importance of perinatal care connections.^24^

Alcohol and drug use have been associated with many adverse health events, both direct (eg cirrhosis, endocarditis) and indirect (eg increased risk of unplanned pregnancy or HIV, and mental health issues).^22^,^25^,^26^ Alcohol is also a particularly risky substance to use in pregnancy, as it can cause fetal alcohol spectrum disorder (FASD), leading to lifelong physical, cognitive, and sensory impairments.^27^ FSW are known to have high rates of alcohol consumption,^28^ although to our knowledge no studies have focused specifically on alcohol use during pregnancy, which is surprising given the high rates of both pregnancy and alcohol use among FSW. Likewise, illicit drug use among FSW has been documented to be higher than the general population,^29^ but no existing studies have looked at drug use among pregnant FSW.

There is a dearth of knowledge about maternal health among FSW, despite the fact that they may be predisposed to higher risk pregnancies due to factors such as ongoing exposure to STI/HIV, violence, and malnutrition secondary to food insecurity.^5^,^18^,^21^ Guidelines on providing healthcare for FSW are lacking, and the need is especially acute in pregnant and postpartum FSW, where a recent scoping review was unable to find any relevant guidelines on maternal healthcare among FSW.^21^ Part of the reason for this is that there is relatively little research on the maternal health of FSW. A recent scoping review of maternal health services among FSW retrieved only 18 studies, conducted in 11 countries; the primary focus of most studies was on uptake of antenatal care.^21^ None of the studies included in that review looked at postpartum depression, substance use during pregnancy, or work habits around the time of delivery.^21^ This study helps to fill these gaps and informs FSW-focused agencies on gaps in care for FSW, barriers to care, and other health-behaviour related information which may help to understand and address root causes behind the high prevalence of maternal deaths found in previous research.

### Study Aims

This multi-country study aims to explore maternal healthcare accessibility and use, including antenatal and intrapartum care, and health risk behaviours during pregnancy, such as substance use and work habits, among FSW in six low- and middle-income countries (LMIC): Democratic Republic of Congo (DRC), India, Indonesia, Kenya, Nigeria, and South Africa. We also aimed to examine barriers to care and to explore FSW suggestions on how to improve reproductive and maternal healthcare for this vulnerable and highly stigmatized population. We hypothesized that FSW would have reduced access to antenatal and skilled intrapartum care and may display behaviours which could further heighten their pregnancy risk, such as substance use.

## Methods

This study is part of a larger study conducted in 2019 designed to examine broad spectrum of maternal and child health needs, health behaviours and outcomes, and causes of mortality among FSW and their children in eight LMIC. The data collection methodology was described in detail elsewhere.^13^ This analysis focuses on the maternal health components of the survey administered in six countries. Two countries from the original study, Angola and Brazil, were excluded from this analysis due to poor data quality on the maternal health sections of the survey resulting from logistical constraints experienced at the time of data collection.

### Study Design, Participant Recruitment, and Setting

This is an observational cross-sectional study. A two-stage non-randomized sampling design was used, first recruiting local partners who provided services to FSW. The lead researcher (BW) contacted these service providers and sent letters of invitation to participate in the study. In the second stage, local partners who agreed to participate recruited study participants. Country selection criteria included: a large FSW population, high national maternal mortality rate, high HIV infection burden among FSW, availability of local partner organizations to assist in recruitment, and geographic diversity.

During the time of data collection, sex work was not decriminalized in most of the study countries. As FSW are a hidden population with no registries or fixed addresses, multiple recruitment methods were used to recruit as many FSW study participants as possible. Local partners used purposive sampling to contact FSW from a variety of places, including brothels, bars, or on the streets, fields or parks. Snowball sampling was also used, as some FSW contacted their peers to participate. Our methodology differs from simple random sampling in that our sample selection used a combination of purposive and snowball sampling methodologies, as a random sample selection among a hidden population group such as FSW without any records of contact information and fixed addresses would not be feasible.

Demographic questions were adopted from the Demographic Health Survey.^30^ Additional maternal health survey questions were designed by the research team based on their existing knowledge of FSW health needs, with input from our local partners. The questions were related to ANC, sex work during pregnancy, location of delivery, substance use in pregnancy, and depression among mothers with young babies (a surrogate for postpartum depression). Local partners reviewed the questionnaire prior to administration and made any necessary adjustments to adapt to the local context. To gather the most information in an efficient manner, a structured questionnaire with a combination of closed and open-ended format was used (Appendix 1). Closed-ended survey questions were formatted as a Likert scale to get input from FSW as proxy respondents using their knowledge of the FSW community to respond to questions about other FSW within their community.

They were asked to respond to these questions by estimating how many of their peers out of a group of 10 would engage in certain behaviours (eg seeking antenatal care, drinking alcohol during pregnancy) by giving a numerical measure of Likert scale ranging from 0 to 10. For determining SBA, participants were asked where most FSW give birth. Responses were then classified into skilled or unskilled birth attendance by the researchers, with input from our local partner organizations.

In this mixed method exploratory study, we also collected qualitative and text data. The goal of the open-ended questions was to gather detailed answers to the questions from the perspective of each participant. Due to time and logistical constraints, detailed qualitative interviews were not held with participants, although participants were given ample time to answer all open-ended questions (Appendix 1).

### Selection of Data Collection Sites and Participants

Local partners recommended cities/towns for recruitment of participants for the discussion groups, based on factors such as diversity of settings (ex. urban vs rural), and where they felt they could recruit enough participants for the study. Local partners also chose the locations for the discussion group data collection sessions in a place they felt was safe and confidential for participants. Local partners screened participants based on the following inclusion criteria: age 18 or older, mother of at least one child aged ≤10 years, engaged in full-time sex work during the preceding three years, and interactive with other FSW in the community. The requirement to have a child was related to the child health component of the questionnaire. Exclusion criteria included participants who were impaired by drugs or alcohol and unable to contribute to the discussion groups, as well as participants who posed a threat to other group participants. Local partners ensured that each woman participated in only one discussion group.

Altogether, 24 cities with a range of 1–7 cities per country were included (Table 1). Overall, 176 groups with 10–12 participants in each group were convened, with 2–15 groups per city and 12–36 groups per country. The variation between countries was due to logistical factors such as distance travelled between locations, ease of recruitment of FSW, and number of FSW in the area available for study participation. A total of 1352 women were interviewed.

**Table 1:** Discussion Group Participation by City and Country

Country	Cities	Number of Discussion Groups (% of All Groups)	Total Number of Participants (% of All Participants)
Democratic Republic of Congo (DRC)	Bukavu	31 (20.0%)	311 (23.0%)
	Kinshasa		
India	Bangalore	20 (14.8%)	152 (11.2%)
	Chennai		
	Gudibanda		
	Hyderabad		
	Nashik		
	Salem		
	Warangal		
Indonesia	Jakarta	12 (8.9%)	71 (5.2%)
Kenya	Kisumu	37 (13.3%)	362 (26.8%)
	Mombasa		
	Nairobi		
Nigeria	Abuja	32 (23.7%)	312 (23.1%)
	Calabar		
	Lagos		
South Africa	Cape Town	26 (19.3%)	144 (10.7%)
	Durban		
	Johannesburg		
	Port Shepstone		
**Total**		**135 (100%)**	**1352 (100%)**

### Data Collection Methodology and Quality Assurance

Discussion groups of FSW were convened and a semi-structured questionnaire of both open- and closed-ended questions was administered. The lead researcher (BW) administered the questionnaire in South Africa, Kenya, and Nigeria. In other countries, the lead researcher trained a staff member from our local partner, who then translated the questions and answers, under the supervision of the lead researcher, who recorded responses. The data were primarily quantitative, but text data in the form of comments and responses to open-ended questions were also recorded. Local partner organizations were provided with a lay summary of their city’s or country’s results for review to ensure no errors or omissions were made, as well as to make their local data immediately available to them.

Questions in the survey were primarily quantitative, closed-ended questions. There were also a number of open-ended questions in the questionnaire (Appendix 1), which were used for the qualitative analysis using text analytics. Due to time and logistical constraints, detailed qualitative interviews were not held with participants, although participants were given ample time to answer all open-ended questions. Participants were asked not to provide personal identifying data. Informed consent was obtained, and participants were compensated with an amount of small cash deemed appropriate by local partners. All answers to the questions were recorded by the survey administrator, but to ensure confidentiality of respondents, all responses were recorded anonymously, and responses amalgamated and analysed by country.

We used the proxy respondent method (PRM) of data collection to estimate the prevalence of phenomena. PRM is commonly used in epidemiologic research.^31^ The PRM has been used to estimate prevalence of stigmatized behaviours, such as substance use, through indirect reporting, which may be more accurate than direct reports, due to minimization of social desirability bias.^32^,^33^ Sheikazad et al used a random sample of “proxy respondents”, peers of college students (“alters”) to examine the risky or stigmatized behaviours, such as alcohol use, and found PRM-based prevalence estimates of the community behaviour is closer to the direct individual estimated average of the community than the community network-based estimates.^33^,^34^ The PRM has been used and validated for data collection of maternity care, including prenatal care, delivery location, and pregnancy and birth outcomes.^35^ Nwaru et al (2012) compared the PRM (close relatives as proxies) estimates with index-subject-based estimates on maternal characteristics, prenatal care, pregnancy, and birth outcomes in rural China and found reasonable accuracy with less non-responses and misclassifications by PRM data.^35^ Our research team has found that FSW often do not live with their biological families but have strong social bonds with other FSW, their “surrogate sisters”, and are knowledgeable about and willing to share their “sisters’” health behaviours and outcomes.^36^ In this exploratory study, we wanted information from as many FSW in a community as possible to provide information about other FSW within their community.

### Data Analysis

The mean of all participant responses was calculated for each country and weighted averages, accounting for the number of participants in each country, were then calculated when responses from all countries were combined. Results of Likert scale questions, with a response range of 0–10, are presented as estimated percentages of FSW. For example, a country with a mean estimate of 3.7/10 represents a 37% prevalence. Literature suggests that Likert scales may be treated as numerical responses and analyzed parametrically.^37^

For the analysis of the open-ended questions, textual responses pertaining to maternal health needs were obtained from participants who answered the question “What can NGOs do to help children of sex workers and their mothers in this community?” (n=254). Maternal health-related comments were extracted using filtering of text data by content. Only some participants provided maternal health-related responses; hence, the textual response sample size is less than the total number of participants. We used the text analytics methodology of text mining using RapidMiner software to analyze participants’ expressed needs related to pregnancy and reproductive health (n=94), as well as to get more detailed information on the place of childbirth. After filtering the contents, the resulting text data were analyzed by categorizing the terms (coding) occurrence and clustering of the codes to identify themes arising from the clusters. Common terms in each cluster were assigned to a thematic category.

## Results

Descriptive summaries of survey data are included in Tables 2–6. Overall, an estimated 86.1% of FSW were mothers, with an estimated average of 3.9 children/FSW, but with a wide range from 1 to 11 (Table 2).

**Table 2:** FSW as Mothers

Country	Estimated Proportion of FSW who are Mothers (%)	Estimated Average Number of Children per FSW (Range)
DRC	82.2%	4.7 (1–10)
India	85.3%	2.4 (1–5)
Indonesia	82.3%	2.2 (1–7)
Kenya	89.0%	3.3 (1–11)
Nigeria	86.7%	4.2 (1–10)
South Africa	89.0%	3.8 (1–7)
**Total average**	**86.1%**	**3.9 (1–11)**

**Table 3:** Maternal Health Behaviours and Characteristics of FSW: Alcohol and Drug Consumption, Reported Sadness/Depression, and Antenatal Care Attendance

Country	Consumed Alcohol during Pregnancy (%)	Consumed Drugs during Pregnancy (%)	Mothers of Young Children who Felt Sad or Depressed (%)	Percent of FSW who Seek Antenatal Care
DRC	81.9	76.4	73.5	11.9%
India	25.2	48.9	45.7	57.4%
Indonesia	66.8	43.4	44.3	43.0%
Kenya	80.5	61.0	65.0	32.6%
Nigeria	85.1	66.9	74.3	21.8%
South Africa	88.8	66.2	75.2	25.7%
**Weighted average**	**79.0**	**65.1**	**70.6**	**28.6%**
**% (range)**	**(25–89)**	**(43–76)**	**(44–75)**	**(12–57)**

**Table 4:** Where Most FSW Deliver-Skilled or Unskilled Attendant

Country	Skilled	Unskilled
DRC	10.7%	89.3%
India	100%	0%
Indonesia	87.1%	12.9%
Kenya	66.5%	33.5%
Nigeria	14.0%	86.0%
South Africa	72.6%	27.4%
**Weighted average**	**42.37%**	**57.63%**

**Table 5:** Estimations of When FSW Stop Working When Pregnant

Country	<3 Months Gestation (%)	4–6 Months Gestation (%)	7–9 Months Gestation (%)	At Onset of Labour (%)
DRC	0%	1.2%	56.6%	41.9%
India	0%	40.5%	59.5%	0%
Indonesia	6.4%	44.4%	38.1%	11.1%
Kenya	0.9%	9.5%	75.8%	13.8%
Nigeria	0%	7.4%	89.6%	3.0%
South Africa	0%	5.3%	28.1%	66.7%
Weighted Average	0.7%	9.7%	65.2%	24.4%

**Table 6:** Estimations of Return to Work After Childbirth

Country	<1 Week	1–4 Weeks	1–3 Months	3–6 Months	>6 Months
DRC	45.8%	40.9%	11.2%	2.1%	0%
India	2.9%	2.9%	26.5%	55.6%	11.7%
Indonesia	12.1%	27.3%	53.0%	6.1%	1.5%
Kenya	17.6%	55.4%	23.8%	2.6%	0.6%
Nigeria	35.4%	46.1%	12.7%	4.9%	1.0%
South Africa	52.2%	33.7%	10.9%	3.3%	0%
**Weighted Average**	**31.7%**	**44.2%**	**18.3%**	**5.0%**	**1.0%**

Estimated access to ANC was an average of 28.6% across all countries (Table 3), ranging from 12% in DRC to 57% in India. For FSW in sub-Saharan Africa, an average of less than 35% of FSW were estimated to seek ANC in any of the countries surveyed.

Text data analytics revealed a variety of reasons for not accessing antenatal care. Lack of money and no health insurance were the major barriers identified by FSW in DRC and Nigeria, the two countries with the lowest uptake of antenatal care. Discriminatory treatment by healthcare workers was the second most common barrier identified by these women in DRC and the primary barrier in Kenya. In Nigeria and South Africa, women identified the inconvenience of accessing healthcare as a major barrier. India was the only country where FSW identified that they were not allowed to access antenatal care by their bosses (eg brothel manager). Participants also indicated a variety of personal reasons for not accessing antenatal care including the perception that it was not necessary, fear of being tested for HIV, revealing pregnancy, not planning to continue the pregnancy, lack of self-esteem, lack of interest, stigma due to absent partner, fatigue, and substance use.

In terms of substance use, an average of 79% of pregnant FSW were estimated to consume alcohol during pregnancy (Table 3). Most countries hovered around 80% alcohol usage during pregnancy, except India, where it was 25%, with the high being nearly 90% of pregnant FSW consuming alcohol in South Africa. Although lower than alcohol use, drug use by pregnant FSW was still estimated to be high, at 65.1% overall, ranging from 43.4% in Indonesia to 76.4% in DRC (Table 3). From text analytics, it was determined that the most common drugs reported included opiates (codeine and tramadol), as well as cannabis, crack, cocaine, khat (a stimulant), methamphetamines, and tobacco.

The average reported prevalence of depression or sadness among FSW with young babies was 70.6% (Table 3). In all sub-Saharan African countries, the reported prevalence of depression or sadness of FSW with young babies was over 65%, while in India and Indonesia, it was slightly lower at about 45%.

Government hospital or midwife-attended births were classified as SBA, although participants reported delivering in a variety of locations. The most common location of delivery in DRC and Nigeria was in the brothel. Other unskilled locations included at home, with traditional birth attendants, or at “local maternity hospitals”, which are operated by untrained birth attendants according to our local partners. There was notable variation in where most FSW were estimated to deliver, ranging from 11% of FSW in DRC and 14% in Nigeria estimating that most FSW had skilled delivery, to 100% of FSW in India stating that they felt that most FSW delivered in locations where SBA was available (Table 4).

Many participants, especially those in sub-Saharan Africa, reported pregnant FSWs continuing to work until near the end of their third trimester, many “up to the last day” (Table 4). There was regional variation, with 42% of FSW in DRC estimated to work until the onset of labor, compared to India and Indonesia, where 41% and 44% stopped work by 6 months. An average of 75.9% of FSW were estimated to return to work at less than a month postpartum (Table 5). In South Africa, over half of FSW were reported to return to sex work within less than a week postpartum, while in India women tended to return to work later, with over half taking at least 3 months off. Text analytics revealed that the primary reason for working so late into pregnancy and returning to work so soon postpartum was economic, with food insecurity being a main driver. Textual analysis also revealed that FSW reported a need for improved housing, especially while pregnant and postpartum.

### Maternal and Reproductive Health Needs of FSW: Text Analytics Results

Analysis of the text data on the reproductive and maternal needs of FSW revealed the need for: 1) health education on safe abortion, postnatal care, safe sex, and nutrition education during pregnancy; 2) safe abortion care; 3) pre- and post-natal care; 4) shelter during pregnancy and postpartum; 5) condom distribution and family planning counselling to avoid unintended pregnancies; 6) mobile clinics, STI testing and checkups and 7) psychosocial supports to help prevent suicide. FSW identified financial stressors and food insecurity for themselves and their children as a significant cause of stress and the main reason why they work so late into pregnancy and start back so early after birth. The main deterrents to seeking maternal and reproductive care were cost, stigma, and accessibility. FSW identified a desire for FSW-friendly and accessible maternal healthcare, such as antenatal care at a local NGO or drop-in center they are familiar and comfortable with, or even at the local “hot-spot” (place where FSW gather) to improve accessibility and eradicate stigma, as well as financial supports to cover the cost associated with receiving services.

## Discussion

Our study revealed multiple risk factors that expose FSW to increased maternal and perinatal risk. Unlike our current study which spans across multiple countries, previous literature on maternal health among FSW is limited, and has generally been limited to a single city or country.

### Antenatal Care

Our study findings confirmed that, like existing studies,^3^,^5^,^13^,^26^ nearly all FSW were reported to be mothers (86%). In our study, FSW estimated that few of their peers received ANC; just over 1/3 on average, although there was notable country-level heterogeneity, ranging from a high of 57% in India to a low of just 12% in DRC. This is far below the WHO goal of 90% pregnant women receiving at least eight ANC contacts.^38^ FSW in our study estimated accessing ANC at much lower percentage than other women in the same country. For example, in Kenya, 90% of all women received at least one antenatal visit,^39^ while in this study Kenyan FSW estimated that only 33% of their peers had access to any antenatal care. The country with the highest reported ANC access at 57% was India; however, this was still much lower than general population averages in sub-Saharan Africa where maternal healthcare access has traditionally been challenging and where 78% of women receive at least one antenatal visit.^16^ Our findings revealed that the major reasons for FSW not accessing ANC were cost, lack of knowledge of the importance of ANC, inaccessibility, and discrimination from healthcare workers. FSW in our study identified a desire for improved and more accessible ANC.

Previous research has reported that the leading causes of death among FSW in LMIC are pregnancy-related.^13^ The current findings suggest that lack of ANC and SBA may have some bearing on maternal mortality in this population of women. Given that ANC reduces maternal and perinatal mortality,^11^ improving vulnerable women’s access to appropriate and respectful ANC may decrease the high maternal mortality among this group. It may also decrease perinatal HIV transmission and increase skilled birth attendance.^16^,^21^ Additionally, ANC is a time for anticipatory guidance around many topics, including substance use. Given the high reported prevalence of substance use in our findings, this is particularly relevant.

The FSW interviewed expressed a desire for accessible, affordable, and respectful ANC. While the barriers to adequate ANC in pregnancy are well known, few solutions have thus far been implemented. Structural interventions to address stigma experienced by FSW, such as educating healthcare providers (HCP) and empowering FSW to address inappropriate treatment by HCP have been advocated by the WHO and shown to be effective in South Africa and India.^40^ Linking HIV services to sexual and reproductive health services, including maternal health services, could also decrease barriers to care.^40^ Drop-in centers and peer educators have also been shown to be preferred by FSW.^13^ In our study, FSW identified having care in a place they feel safe and comfortable, such as the hot spot, as a priority, which could increase ANC uptake by these vulnerable women. Focusing solely on HIV prevention and condom use, or even adequate contraceptive access, risks missing the needs of the many FSW who become pregnant, irrespective of whether the pregnancy is desired.^10^

### Safe Delivery

Estimates as to where most FSW give birth showed marked variation between countries. FSW in India had universal agreement that most FSW give birth in SBA locations. By contrast, less than 15% of FSW in DRC and Nigeria thought that most FSW delivered in a location with a skilled attendant; the most common location of delivery reported in both countries was in the brothel. Other sites identified as locations where most FSW give birth included home, the hot spot, unlicensed private facilities lacking trained personnel, or in the street or field.

Delivery outside of hospital settings is risky for many reasons. Women delivering in these locations are at risk of increased maternal and neonatal morbidity and mortality due to, among other things, the lack of skilled birth attendants, hygienic space, and essential medications. Even low-risk pregnancies can become high-risk deliveries, and the lack of skilled birth attendants is a key risk factor for maternal mortality, as delivery is the highest-risk time for both mother and baby.^16^ If complications arise when FSW are delivering unattended in a brothel or other non-healthcare facility, there is often no money or time to transport them to a medical facility, further increasing the likelihood of both maternal and neonatal morbidity and mortality.^41^

Interestingly, both Nigeria and DRC reported by far the lowest level of ANC and skilled birth attendance compared to other countries studied. ANC has been linked to an increased chance of having a skilled birth attendance,^16^,^17^ so increasing ANC in women in these countries may be key to increasing skilled birth attendance, although there could be other factors, such as cost or stigma, that could be contributing to both; further study is warranted.

The high number of maternal deaths among FSW in these countries has been previously documented.^13^ Unsurprisingly, the country with the most maternal deaths, DRC, was also the country with the lowest uptake of antenatal care by FSW (12%) and the lowest estimates of SBA (11%).

### Sex Work During Pregnancy and Return to Work After Delivery

Continued engagement in sex work during pregnancy is an added risk factor that we found in this study that we did not find reported elsewhere. This study confirms that FSW often work until the end of pregnancy, although there was a large inter-country discrepancy in terms of how long pregnant FSW continued working; overall, nearly ¼ (24.4%) of FSW were estimated to work until the onset of labor, with 90% working past 7 months’ gestation. Risks of continuing to practice sex work throughout pregnancy include ongoing exposure to harm including STIs, including HIV,^5^ substance use, as well as violence and trauma,^4^,^5^ which can lead to abruption, miscarriage, preterm labor, or fetal demise.^42^

There has been variability in the literature about timing of return to work after delivery.^5^,^21^ Our data indicate a very short return to work interval after giving birth, with 76% of FSW estimated to return to work less than one month after delivery, and 31% returning within less than a week. This is well under the 6–8 week timeframe that it takes the body to fully heal and return to its pre-pregnancy state.^43^ Going back to work within the first few days postpartum may put FSW at increased risk of acquiring STIs, as unhealed vaginal trauma may increase the risk of STI if there is exposure. Such a quick return to work can also disrupt breastfeeding, which may be detrimental to the newborn’s health.

In this study, FSW identified financial stressors and food insecurity as the main reasons for working late into pregnancy and returning to sex work soon after delivery. Ironically, if breastfeeding is disrupted by an early return to work, these women may have to supplement with formula, which is an added expense for these food-insecure women. The FSW in our study also identified a desire to have a safe place to stay during pregnancy and recuperate postpartum.

### Substance Use During Pregnancy

While high rates of alcohol use among FSW has been documented elsewhere,^26^,^28^,^44^,^45^ our finding that 79% of FSW are estimated to consume alcohol during pregnancy is, to our knowledge, a novel finding, and one that is very concerning, given that alcohol is a known teratogen that can cause FASD.^27^ As with some of the other metrics, India was an outlier, with only 25% estimated to consume alcohol in pregnancy, which is still concerningly high, but lower than reports by FSW in the other countries surveyed where the majority of FSW were reported to drink alcohol while pregnant. The rate of alcohol consumption in all sub-Saharan African countries studied was estimated at over 80%. Reasons for the higher reported rates of alcohol use in sub-Saharan Africa were not explored and could be the topic of further study.

Similarly, the finding that our proxies reported an average of 65% of FSW over six countries used recreational drugs during pregnancy is alarmingly high, and to our knowledge the first published statistic of its kind. Although alcohol is the most concerning teratogen amongst recreational substances, other substance use in pregnancy can also be harmful for both mother and fetus. Most recreational drugs have been associated a variety of adverse obstetrical outcomes such as low birth weight, preterm birth, and abruption, which can be life threatening to the fetus as well as the mother.^45^ Many recreational substances have also been associated with cognitive issues for children exposed in-utero and other health issues for the users.^45^ In our study, substance use further added to FSWs’ obstetrical risk, as it was cited as a reason that women sometimes failed to attend ANC.

Further studies should examine alcohol and other drug use patterns among FSW, especially during pregnancy, and look for evidence of FASD among children of FSW. In addition to further studies, programs targeting increased awareness of the harms of alcohol and drugs during pregnancy and decreasing alcohol and drug use among pregnant FSW, as well as supports for affected children, are urgently needed. This would ideally be integrated into targeted ANC for FSW, as it is a practical time to address these issues, and the earliest possible cessation of substance use in pregnancy is ideal.

### Postpartum Depression

The reported rates of depression or sadness among mothers with young babies in our study was on average 71%, with a range of 44–75%, depending on the country. Once again, India and Indonesia both had a relatively lower prevalence around 45%, with the remaining four sub-Saharan African countries all averaging over 65%. Even the lower numbers reported in India and Indonesia are much higher than global reported rates of postpartum depression of about 10%.^23^ It is also much higher than reported rates of depression among general surveys of FSW in LMIC, which range from about 20–40%.^22^,^46^ In our previous study, suicide in pregnancy or postpartum was the second most frequent cause of maternal deaths among FSW after abortion,^13^ and 62% of all FSW suicides were maternal suicides; most of these (58%) occurred during pregnancy.^24^ Given the high reported prevalence of sadness or depression after giving birth, combined with our previous finding of high numbers of maternal suicides, there is an urgent, unmet need for mental health services for pregnant and postpartum FSW, a need also identified by FSW themselves in this study.

## Future Directions

Ultimately a rights-based approach with the creation of targeted services that are acceptable, affordable, timely, and appropriate is needed.^13^ Given the significant regional variation we found, an individualized, community-by-community approach should be taken. Further studies at the local and national levels could be useful in further elucidating the exact maternal health needs of FSW and the best way to address them. However, certain broad principles apply to pregnant FSW in all countries studied: they have high levels of drug and alcohol use in pregnancy, they experience a range of barriers to quality ANC and safe delivery, and seem to be at higher risk of depression postpartum.

Options to address these challenges could include peer mentorship and community empowerment.^13^ A variation of the mothers2mothers mentorship program, which was designed for mothers with HIV, could be designed for pregnant FSW, as it helps stigmatized women seek care and garner support from other women who understand their situation.^5^ Global Health Promise has a started a unique program promoting maternal health among FSW and sexually exploited adolescent girls, operating in Uganda, Kenya, and Nigeria, ensuring they receive quality ANC and SBA. Ultimately, the siloes between HIV care, mental health care, and maternity care need to be broken down, and stigma against FSW, especially the societal aversion to the idea of FSW as mothers, needs to be addressed if we are to reduce maternal mortality (SDG target 3.1) and improve health outcomes for all women.^10^ The women interviewed clearly identified that there are many social determinants of health beyond just medical care that are affecting their health, so focusing on maternal health services alone, while important, is unlikely to be sufficient.

## Strengths and Limitations

This is an exploratory study and brief overview of maternal health behaviors and characteristics of FSW in six LMIC. Strengths of this study include its exploratory nature in broadly looking at pregnant and postpartum FSW, an understudied population, and encompassing a wide variety of maternal health issues to identify key risk factors for further study. We talked directly to the women who had lived experience with sex work. Additionally, multiple sites and countries were surveyed, showing both similarities and differences among maternal health issues for pregnant FSW across different countries and cultures. Using the PRM may lead to more accurate reporting, as asking participants directly about stigmatized behaviors may lead to under-reporting, due to social desirability bias.^32^,^33^ Thus, the PRM has two principal advantages: firstly, it avoids social desirability bias and risking FSW either being untruthful due to shame or outing them by having them admit potentially stigmatizing events or behaviours in front of their peers. Secondly, it gets a broader picture of what patterns are occurring in the community rather than individual experiences. Congruence of our data collected by the PRM with other studies, such as the high proportion of FSW who are mothers, provides assurance to the validity of findings by proxy respondents.

Triangulating data about FSW can be challenging, as many of these women do not access formal healthcare settings, where records could be reviewed. However, many comments and themes were found across and between groups (ex. common barriers to antenatal care, location of delivery, substance use during pregnancy, etc), adding to the credibility and trustworthiness of this data. Our mixed-method study provided methodological triangulation and also context and depth to the trends revealed by the quantitative aspects of the questionnaire.

This study found substantial inter-country heterogeneity, indicating that further studies should be used to discover specific needs of FSW locally to drive country-specific policy and programmatic changes. We did not conduct in-depth qualitative interviews, the use of which could lead to richer data and explanations for some of the trends we have noted. However, we did include a number of open-ended questions, which allowed for discussions among the women and for them to raise any concerns or ideas that may not have been addressed in the closed-ended questions.

While they have been validated for a variety of scenarios in epidemiologic research and may lead to more accurate reporting in the context of stigmatized topics,^32^,^33^ proxy respondent methods can be subject to misclassification through loss of precision (random measurement error).^31^ However, we chose to use the PRM due to the risk of social desirability bias if questions were asked directly of participants, especially given the sensitive nature of questions asked.

For the SBA question, our numbers reflect an overall estimation of where “most” FSW deliver according to community peers who participated in the study. We also did not specify the meaning of “most”, whether it meant nearly all or just a simple majority. The question was intentionally phrased this way to allow an exploration of the various locations where “most” FSW in their community deliver and thus, identify trends and areas of need within the limited scope of the survey.

On the questions about substance use in pregnancy, we did not clarify whether it was “ever use” versus “regularly use,” nor did we ask about the frequency or amount of substance use during pregnancy, as this was designed as an exploratory study to determine if substance use among pregnant FSW was an issue. Similarly, our ANC question was phrased as a binary, ie, whether care was sought, yes or no; further details on timing and frequency of visits were not explored, though it would be useful in guiding interventions.

## Conclusion

This exploratory study of FSW maternal health reveals many factors that contribute to FSW pregnancies being high risk. Pregnant FSW have multiple, overlapping risk factors for adverse obstetrical outcomes, including low ANC attendance, low SBA rates, high rates of brothel and hot spot delivery, high prevalence of substance use in pregnancy, working late into pregnancy and returning to work shortly after birth, and high rates of postpartum depression. The high number of maternal deaths^13^ and adverse perinatal outcomes among FSW may be attributable to the risk factors identified in this study. Although heterogeneity exists between countries, these risk factors were present in all six study countries. Stigma as well as other social determinants of health play a key role in the risk factors experienced by pregnant FSW. Targeted services should be provided to not only increase ANC and SBA uptake but to decrease substance use in pregnancy, and address the social determinants of health to improve FSWs’ maternal health.

## Data Availability

The first author, HT, affirms that the manuscript is an honest, accurate, and transparent account of the data collected, stored, and analyzed. Due to ethical considerations (ie, consent was not obtained or given for open data access or additional data usage), controlled and secure data access and usage is necessary for subject protections. De-identified aggregate data used for this analysis can be requested from the corresponding author. Access permission will be considered based on the following usage criteria: (a) for the purpose of partnering on research on female sex workers; (b) for inclusion in curriculum for educational purposes; or (c) for the provision of services to female sex workers and their children by governmental and non-governmental organizations.

## Abbreviations

ANC, Antenatal care; DRC, Democratic Republic of Congo; FASD, Fetal alcohol spectrum disorder; FSW, Female sex worker; HCP, healthcare provider; LMIC, Low and middle income country; PRM, Proxy respondent method; SBA, Skilled birth attendance; WHO, World Health Organization.
